# Molecular Mechanisms of Glucose Fluctuations on Diabetic Complications

**DOI:** 10.3389/fendo.2019.00640

**Published:** 2019-09-18

**Authors:** Zhen-Ye Zhang, Ling-Feng Miao, Ling-Ling Qian, Ning Wang, Miao-Miao Qi, Yu-Min Zhang, Shi-Peng Dang, Ying Wu, Ru-Xing Wang

**Affiliations:** Department of Cardiology, Wuxi People's Hospital Affiliated to Nanjing Medical University, Wuxi, China

**Keywords:** glucose fluctuations, diabetes, reactive oxygen species, protein kinase C, protein kinase B, mitogen-activated protein kinase

## Abstract

Accumulating evidence indicates the occurrence and development of diabetic complications relates to not only constant high plasma glucose, but also glucose fluctuations which affect various kinds of molecular mechanisms in various target cells and tissues. In this review, we detail reactive oxygen species and their potentially damaging effects upon glucose fluctuations and resultant downstream regulation of protein signaling pathways, including protein kinase C, protein kinase B, nuclear factor-κB, and the mitogen-activated protein kinase signaling pathway. A deeper understanding of glucose-fluctuation-related molecular mechanisms in the development of diabetic complications may enable more potential target therapies in future.

## Introduction

Diabetes is a chronic metabolic disorder and a threat to human health in the world (1). In the past decades, it has been verified in both clinical and experimental studies that glucose fluctuations are more harmful than in a comparative state of constant hyperglycemia (2). Recently, increasing levels of experimental researches have investigated the relationship between glucose fluctuations and diabetic complications (3). This review is focused on describing the variety of underlying molecular mechanisms influencing glucose fluctuations on diabetic complications in different tissues and organs.

## Overview and Diagnostic Indicators of Glucose Fluctuations

With the development of new technologies such as continuous blood glucose monitoring systems, measurements of blood glucose fluctuations have become increasingly accurate, providing new research directions for clinical and basic studies (4). The standard deviation of the mean around the mean glucose value is a golden standard to measure glucose variability (5). However, the mean amplitude of glycemic excursions index is more advanced and also appropriate (6). Commonly used indicators include (Table 1): (1) mean amplitude of glycemic excursions (7); (2) standard deviation of the mean (8); (3) coefficient of variation (9); (4) glycemic lability index (10); (5) mean of daily difference (11); (6) continuous overlapping net glycemic action (12); (7) high blood glucose index and low blood glucose index (12).

**Table 1:** Commonly used indicators for blood glucose fluctuations.

**Concept**	**Definition/Calculation**	**Advantage**
Mean amplitude of glycemic excursions (MAGE)	The mean of blood glucose values exceeding one SD from the 24-h mean blood glucose.	The most commonly used term as an indicator of blood glucose fluctuations.
Standard deviation of the mean(SD)	Standard deviation of patient's mean blood glucose level in a certain period of time.	It represents the change and dispersion rate of the average blood glucose concentrations.
Coefficient of variation(CV)	SD/mean × 100%.	It normalizes glycemic variability at different mean blood glucose values.
Glycemic lability index(LI)	It processes three glucose values to calculate a lability value and then moves to the next three glucose values, and so on.	It can serve as an indicator of patients' prognosis.
Mean of daily difference (MODD)	It is calculated as the average of the difference between values on different days but at the same time.	It can be used to assess the continuous changes of blood glucose between different days.
Continuous overlapping net glycemic action (CONGA)	It is calculated by determining the difference between values at different set intervals, and the difference is then applied to the CONGA formula.	It is a parameter that reflects the variability of blood glucose over a certain time interval.
High blood glucose index (HBGI) and low blood glucose index (LBGI)	They are implemented by converting glucose values into risk scores. If the glucose risk score is below 0, then the risk is labeled as LBGI, and if it is above 0, then it is labeled as HBGI.	They can assess the risk of severe hypoglycemia or hyperglycemia in diabetic patients.

*See text for references*.

## Relationship Between Glucose Fluctuations and Diabetic Complications

### Glucose Fluctuations and Microvascular Complications

Glucose fluctuations can lead to chronic kidney disease characterized by progressive albuminuria eventually leading to end-stage renal failure in type 2 diabetes (13). Several clinical studies conducted in patients with type 2 diabetes have indicated that glucose fluctuations may induce a decrease in glomerular filtration rate and increase the risk of chronic kidney disease (14). In addition, it has been observed in animal-based research and modeling of mice and rats with both two types of diabetes that glucose fluctuations can alter renal tissue structure, promote renal fibrosis, and thicken capillary basal membrane (15, 16).

Diabetic retinopathy (DR) is a microvascular complication, which is considered as one of the main causes of visual impairment and blindness (17). DR-related glucose fluctuations can lead to the gradual onset of neurodegeneration and structural damage of the retina, ultimately causing blindness in patients with type 1 diabetes (18). However, whether or not fasting plasma glucose variability is a risk factor that can be used to predict the occurrence of DR is controversial (19). It is commonly accepted that intensive glycemic control can reduce the incidence and damage of DR (20).

Glucose fluctuation is an important risk factor in the morbidity of diabetic neuropathy, including peripheral and autonomic neuropathy (21). Several retrospective longitudinal studies conducted on patients with type 1 diabetes found that glucose fluctuations might be a contributing factor in development of diabetic peripheral neuropathy (22), as well as cardiovascular autonomic neuropathy (23). Diabetic neuropathy can cause impairment of cardiac autonomic modulation, which can be alleviated by controlling undesirable glucose fluctuations, especially in women with type 2 diabetes (24). However, Siegelaar et al. (25) contrastingly found that glucose fluctuation was not involved in the development of autonomic and peripheral neuropathy in patients with type 1 diabetes. Therefore, the relationship between glucose fluctuations and neuropathy in diabetic patients still needs further study.

### Glucose Fluctuations and Macrovascular Complications

In addition to microvascular complications discussed above, glucose fluctuations can also have detrimental effects on vessels, leading to cardiovascular, cerebrovascular, and peripheral vascular diseases. Many cardiovascular complications are associated with glucose fluctuations, including heart failure (26), and arrhythmia (27). The relationship between coronary heart disease and glucose fluctuations is antagonistic such that the latter can gradually cause coronary plaque accumulation (28). Further, patients with type 2 diabetes and coronary heart disease have larger glucose variability than those without coronary heart disease (29). Additionally, more serious glucose fluctuations may lead to higher risk of cerebral infarction in patients with type 2 diabetes, accompanying with worse prognosis (30). Moreover, cardiovascular events may increase in patients with acute ischemic stroke and glucose fluctuations (31).

### Glucose Fluctuations and Cognitive Impairment

In addition to the vascular complications, the relationship between glucose fluctuations, and cognitive dysfunction in diabetics also should not be ignored. Kinoshita et al. (32) evaluated glycoalbumin (GA)/hemoglobin A1c (HbA1c) ratio, Hasegawa dementia scale-revised score and mini mental state examination score of elderly type 2 diabetics and found that GA/HbA1c ratio independently determined the degree of cognitive impairment. Clinical research of Cui et al. (33) have found that gray matter atrophy induced by glucose fluctuations might be responsible for cognitive decline.

## Possible Mechanisms Mediated by Glucose Fluctuations

### ROS Involved in the Regulation of Diabetic Complications

Reactive oxygen species (ROS), as byproducts containing oxygen, are generated through multiple cellular, and physiological mechanisms. ROS contain free radicals including superoxide (•${\text{O}}_{2}^{-}$), hydroxyl radical (•OH), and hydrogen peroxide (H_2_O_2_) (34). Nicotinamide adenine dinucleotide phosphate (NADPH) oxidases, mitochondrial sources and uncoupled nitric oxide synthases (NOS), lipoxygenase, cyclooxygenase, and xanthine oxidase are the major sources of ROS production (1). Excessive generation of ROS plays a significant role in various kinds of physiological and pathological processes (1). Effects of ROS overproduction include the oxidation of DNA, lipid membranes, and proteins (35). Moreover, ROS may also activate oxidative stress-related downstream pathways and lead to indirect cellular damage (36).

#### The Increase of ROS Induced by Glucose Fluctuations

There are relatively few clinical studies that have examined whether glucose fluctuations can cause increased ROS production in diabetic patients. Hoeldtke et al. (37) proposed that excessive oxidative stress correlated with insulin requirements and may lead to β-cell damage in early-staged type 1 diabetic patients. Urinary excretion rate of free 8-iso prostaglandin F2α is an indicator for lipid peroxidation and apparently also may be increased in type 2 diabetic patients with blood glucose fluctuations (38). Compared with chronic sustained hyperglycemia, acute glucose fluctuations seem to be a triggering effect more specifically linked to increased ROS levels (39). Ohara et al. (40) demonstrated that improvements in glycemic variability may reduce oxidative stress in patients with type 2 diabetes. However, a clinical study conducted by Wentholt et al. (41) showed no relationship between ROS and glucose fluctuations in a cohort of type 1 diabetic patients. Correspondingly, more clinical studies are needed to explore the potential relationship between ROS and glucose fluctuations for both type 1 and type 2 diabetic patients.

#### Impact of Glucose Fluctuations on the Antioxidant Defense System

The generation of ROS and scavenging effect of the antioxidant defense system jointly maintain body homeostasis. The antioxidant defense system exerts a restorative function through reversing protein oxidation and damage. The major endogenous antioxidants include superoxide dismutase (SOD), catalase, glutathione peroxidase (GPx), thioredoxin (Trx), vitamin E, and Coenzyme Q10 (42).

The excessive harmful H_2_O_2_ and •OH are rapidly removed via various antioxidant systems. SOD converts •${\text{O}}_{2}^{-}$ to O_2_ and H_2_O_2_. Catalase converts H_2_O_2_ to oxygen and H_2_O. GPx converts hydroperoxides to H_2_O and alcohols (42). The •${\text{O}}_{2}^{-}$ is released from NADPH oxidase, the mitochondrial respiratory chain and eNOS uncoupling, thus, SOD plays a critical role in maintaining oxidative balance (43). Hyperglycemia reduces SOD levels and impairs heart (44), renal (45), and brain (46). Moreover, SOD levels have been reported to be reduced to a greater extent in glucose fluctuations than persistent hyperglycemia (16, 47). GPx1 is expressed most abundantly among GPx isoforms and involved in the detoxification of H_2_O_2_ (48). GPx1 levels are decreased in diabetic rats and the deficiency of GPx1 accelerates atherosclerosis by enhancing pro-inflammatory and pro-fibrotic mechanisms in cardiovascular system (49). Both GPx and catalase levels are reduced in diabetes (50). However, the changes of GPx and catalase levels in diabetes with glucose fluctuations are still unclear.

The Trx system helps to prevent oxidative damage through peroxiredoxins on the setting of physiological conditions (51). Trx seems to function as a protective factor under high glucose conditions (52). Persistent hyperglycemia impairs Trx activity in various target organs, including heart (53), renal (54), and brain (55). However, few data are found in the relationship between glucose fluctuations and Trx. Trx interacting protein (Txnip), a cytosolic protein, binds thioredoxin, and inhibites thioredoxin function. Txnip expression has been reported to be apparently higher in glucose fluctuation group than that in uncontrolled diabetic group (27). The overexpression of Txnip is thought to play an important role in apoptosis of cardiomyocytes and helps to negatively regulate cardiac hypertrophy. Fang et al. (56) found that Txnip overexpression was induced by hyperglycemia through ROS/MEK/MAPK signaling pathway. Along with the increased the processes related to the ROS levels, upregulation of Txnip may be a potential mechanism underlying cardiac apoptosis, and fibrosis in response to glucose fluctuations.

Vitamin E is a lipid-soluble antioxidant and vitamin C is a water-soluble antioxidant. Vitamin E helps to protect the cell by inhibiting lipid peroxidation (57). Vitamin C reacts with •${\text{O}}_{2}^{-}$ and •OH to form the dehydroascorbic acid (58). Hyperglycemia leads to the reduction of vitamin E and vitamin C even their dietary intake is normal (59). Coenzyme Q10 functions as an electron transfer intermediate to add two protons and two electrons to ubiquinone in respiratory chain, which prevents lipid peroxidation. Coenzyme Q10 levels have been reported to be reduced in diabetes (60). However, the effects of glucose fluctuations on vitamin E, vitamin C and coenzyme Q10 levels need further researches.

#### Damages From ROS Overproduction

Diabetes has been reported to be linked to an increase in ROS generation and/or a reduction in antioxidant defenses. Hyperglycemia leads to the ROS overproduction through mitochondrial respiratory chain enzymes, NOS, xanthine oxidases, lipoxygenases, and peroxidases (1). The metabolic route of diacylglycerol—protein kinase C (PKC)—and NADPH-oxidase—ROS generation is enhanced in diabetes (61). NADPH oxidase has been reported to be activated by glucose fluctuations to a greater extent than persistent hyperglycemia and contributes to the increase of ROS production (62). High levels of ROS induced by both constant high glucose and glucose fluctuations lead to different types of cell damages via different mechanisms and eventually permanent tissue damage.

##### The injury of beta cells

Pancreatic islet beta cells (β-cells) play an important role in the balance of glucose homeostasis through regulating insulin secretion in response to changes in blood glucose concentrations (63). Various quantitative indices have been used to appropriately assess β-cell function, including HOMA2%B index, and plasma C-peptide during mixed-meal tolerance test (64). Glucagon is secreted from islet alpha-cells and controls glucose homeostasis in the fasting state (65). A recent clinical study showed that with the extension of glucose fluctuations in type 1 diabetics, glucagon secretory function was gradually impaired (66). Several experiments *in vitro* have reported that glucose fluctuations lead to significant impairment of β-cell function (67). Moreover, evidence from several clinical studies shows glucose fluctuations are involved in β-cell dysfunction (68, 69). Various strategies of blood glucose control can recover β-cell dysfunction which is impaired by glucose fluctuations in patients with both type 1 and type 2 diabetes (70, 71). Glucose fluctuations seem to aggravate β-cells dysfunction more seriously than persistent hyperglycemia (63). Glucose fluctuations have been reported to induce excessive formation of ROS, inflammatory cytokines, and oxidative stress, contributing to the apoptosis of β-cells (72). The β-cell apoptosis induced by both persistent hyperglycemia and glucose fluctuations is closely is closely related to the balance between pro-apoptotic Bcl-2 proteins and anti-apoptotic Bcl proteins at the mitochondrial membrane (73, 74). Hyperglycemia has been reported to induce β-cell apoptosis through various signaling pathways, which is coupled with plasma membrane depolarization through the closure of K^+^ channel and opening of Ca^2+^ channel in response to increased generation of adenosine triphosphate in type 2 diabetes (73). Moreover, Kim et al. (74) proposed that the glucose fluctuations-mediated downregulation of the anti-oxidative enzyme Mn-SOD and anti-apoptotic signal Bcl-2 may contribute to β-cell apoptosis. However, the precise molecular mechanisms by which glucose fluctuations induce β-cell apoptosis than persistent hyperglycaemia remains still unclear. Salvianolic acid B, as a component of Salvia miltiorrhiza, is reported to inhibit INS-1 cell apoptosis induced by intermittent high glucose. Salvianolic acid B can improve Bcl-2 family protein expression and preserve mitochondrial membrane potential by reducing oxidative stress. These findings suggest a potential clinical application of Salvianolic acid B in preventing the ongoing of β-cell dysfunction (75).

##### The inflammation and apoptosis of cardiomyocytes

ROS is a promoting factor for cardiac structural remodeling due to its influence in activating the TGF-β-mediated profibrotic signaling pathway, and in aggravating apoptosis, and inflammation (76). Shotaro et al. (27) established quantitative chronic blood glucose fluctuation models by experimentally fasting streptozotocin-induced type 1 diabetic rats for 24 h and injecting them with regular insulin additionally. Their results demonstrated that ROS levels were significantly higher in response to the induced glucose fluctuations than when compared to values for control treatments with persistent high glucose. High ROS levels contributed to the increase of TNF-α expression and TGF-β1 expression, leading to increased inflammation, and apoptosis of cardiomyocytes (27). Consequently, the incidence of atrial fibrillation was apparently increased due to cardiac fibrosis and apoptosis in diabetic rats with glucose fluctuations (27). Ying et al. (77) held a similar view that cardiac fibrosis and oxidative stress were aggravated in diabetic rats exposed to glucose fluctuations. Further, high level of ROS can induce cardiomyocyte apoptosis via the activation of Bcl-2-associated death promoter regulated by AKT. Along with increased ROS, inflammation and cardiac TNF-α were also increased more obviously in the experimental glucose fluctuation treatment rats, and both were involved the pathogenesis of diabetic complications (77). Thus, restoration of the balance of redox may be crucial in the treatment of cardiovascular complications.

##### Endothelial dysfunction

Glucose fluctuations can induce increased ROS generation in porcine iliac endothelial cells when compared to persistent high glucose, involved in the development of vascular complications (78). Increased endothelial oxidative stress induced by acute hyperglycemia may be related to concurrent activation of NADPH oxidase and consequential superoxide generation (79). Piconi et al. (80) proposed that both stable and oscillating blood glucose could increase oxidative stress and endothelial cell apoptosis through ROS overproduction resulting in the mitochondrial transport chain. In human retinal endothelial cells, the exposure to glucose fluctuations induces the overproduction of ROS at the mitochondrial transport chain level, leading to the development of DR. In this pathological process, over-expression of vascular endothelial growth factor (VEGF) due to excessive endothelial oxidative stress enhanced retinal endothelial cell proliferation (81). *In vivo*, Otsuka et al. (82) injected glucose via the femoral vein into rats after 12 h fasting in order to create a rapid rise of blood glucose. Experimental results revealed that reversible increases in monocyte adhesion were promoted by a temporary rise in blood glucose levels. A clinical study showed the consistent results that blood glucose fluctuations were consistently more likely to increase the vascular endothelial dysfunction in patients with type 2 diabetes compared with a group with persistent hyperglycemia by way of measuring the reactive hyperemia index, an index of vascular endothelial function (83).

##### Neuronal damage

As a member of the mitochondrial anion carrier protein family, uncoupling protein 2 (UCP2) plays a pivotal role in physiological and pathological adaptations of the nervous system (84). UCPs regulate mitochondrial homeostasis by helping to decrease mitochondrial ROS production as well as increasing mitochondrial proliferation and adenosine triphosphate production (85). Cardoso et al. (86) suggested that UCP2 influenced neuronal effects mediated by glucose level fluctuations and deemed UCP as a promising therapeutic strategy. Glucose transporters are located in the brain and involved in bidirectional processes of glucose transport (87). Mismatches between altered glucose transporters and acute glucose fluctuation events can contribute to the neuronal damage. During this process, oxidative stress plays a significant role in the pathophysiological changes in glucose neurotoxicity. Hence, Xie et al. (88) hypothesized that modulating hypoglycemia may improve neurological diabetic complications.

### Inflammation Involved in the Regulation of Diabetic Complications

Inflammation is the primary physiological immune system reaction and plays an important role in the development and progression of diabetic complications (89). The activation of infiltrating inflammatory cells subsequently activates the cells of immune system and promotes the production of inflammatory mediators (90). The release of inflammatory mediators such as cytokine can be mediated by hyperglycemia and oxidative stress (91). Chronic inflammation and oxidative stress are inextricably linked via complex interactions of both mutual amplification in the pathophysiology of diabetes (92).

Moreover, glucose fluctuations have been reported to promote inflammation to a greater extent than persistent hyperglycemia (93). Quincozes-Santos et al. (94) proposed that glucose fluctuations in astroglial cells contributed to consequent cellular biomolecular damage by increasing oxidative stress and pro-inflammatory cytokine release.

### Signaling Pathways Implicated in Glucose Fluctuations

**Figure 1:**
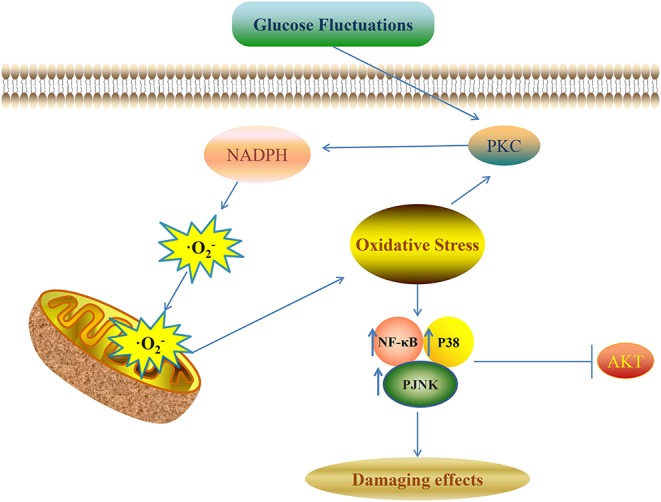
Glucose fluctuations-related signaling pathways involved in development of diabetic complications NADPH aggravates the production of ROS and oxidative stress though a vicious feed-forward cycle. ROS overproduction is the vital trigger factor to activate the downstream signaling pathways and finally leads to various kinds of damaging effects in different target cells and tissues.

Various kinds of signaling pathways are involved in the pathological process of diabetic complications induced by glucose fluctuations. These signaling pathways include protein kinase C (PKC), protein kinase B (AKT), NF-κB, and the mitogen-activated protein kinase (MAPK) signaling pathway (Figure 1) (1). Oxidative stress and pro-inflammatory mediators can be trigger factors to activate these pathways (92).

#### Activation of PKC

PKC belongs to a family of protein kinases with more than 10 isoforms that have exerting functions as downstream targets for lipid second messengers. Overproduction of ROS through PKC-dependent activation of NADPH oxidase in endothelial cells leads to the enhancement of endothelial cell apoptosis (95). Rats with type 2 diabetes were fed twice daily to induce repetitive postprandial glucose spikes and repetitive postprandial glucose fluctuations induced more monocyte adhesion to endothelium than stable hyperglycemia in aortic endothelium and the reduction of postprandial hyperglycemia could effectively improve these changes (96). Adhesion molecules function as a “molecular switch” that regulates the interaction between leukocytes and endothelium, and which is important in pathogenesis, and progress of atherogenesis. In particular, intracellular adhesion molecule-1 and vascular cell adhesion molecule-1 play significant roles in regulation of endothelial cell function (97). Glucose fluctuations can promote overproduction of adhesion molecules by activating PKC-β, completely dependent from mitochondrial superoxide over-production in human umbilical vein endothelial cells (98). Na et al. (99) suggested that PKC-βII membrane translocation increased in endothelial cells with acute glucose fluctuations (100). The activation of JNK can be mediated by PKC-βII and plays an important role in apoptosis of endothelial cells (101). PKC-βII/JNK signaling pathways help regulate endothelial functions through mediating the downstream factors of cytokines and insulin signaling. Hence, the inhibitors of PKC and JNK may effectively reverse endothelia dysfunction induced by acute glucose fluctuations and should be considered further having potential for helping to prevent cardiovascular diseases.

Increased excessive superoxide formation in mitochondria can induce DNA damage by activating PKC and ataxia-telangiectasia mutated proteins (102). Schisano et al. (103) demonstrated that oscillating glucose up-regulated phospho-γ-histone H2AX and PKC-δ more effectively than constant high glucose, consequently leading to p53 phosphorylation. Activation of p53 after exposure to glucose fluctuations up-regulated the expression of genes involved in apoptosis, and seemed to be a more detrimental compared with a state of persistent hyperglycemia (104).

#### Activation of AKT

AKT, as a pivotal signal transduction intermediate, is involved in the pathways of development of diabetic complications (105). Type 1 diabetic rats with fluctuated blood glucose were regularly injected with insulin (6 a.m., 12 p.m., and 6 p.m.) and glucose (9 a.m., 3 p.m., and 9 p.m.) over 12 weeks and the production of ROS was apparently enhanced due to blood glucose fluctuations and related to renal injury (16). Moreover, reduction of phosphorylated AKT leads to activation of glycogen synthase kinase-3β (GSK-3β), which is an initiator of cell death (106). Phosphorylated GSK-3β can activate NF-κB and caspase-3, leading to cardiomyocyte apoptosis (107). Wang et al. (108) proposed that glucose fluctuations can lead to oxidative stress injury in human umbilical vein endothelial cells through PI3K/AKT/GSK-3β pathway. Panax quinquefolius saponi (PQS), known as a Chinese herb, is widely used for diabetes treatment. PQS can reduce oxidative damage induced by glucose fluctuations via the PI3K/AKT/GSK-3β signaling pathway, which provides a potential treatment for improving diabetic cardiovascular complications (108). The AKT signaling pathway is also involved in insulin-dependent glucose uptake and utilization (109). Shao et al. (110) found that intermittent hyperglycemia can aggravate damage to tINS-1 cells more seriously than persistent hyperglycemia by suppressing insulin signaling. Phosphatase and tensin (PTEN), which is located on chromosome 10 plays an important role in regulating cell apoptosis by dephosphorylating PI3K-produced IP3. AKT phosphorylation is dependent on IP3 directly. PTEN expression is increased in the condition of glucose fluctuations and leads to the decrease of p-AKT levels, mediating the β-cells injury. Biscetti et al. (111) indicated that oxidative stress played a critical role in the pathogenesis of intermittent-hyperglycemia-related endothelial dysfunction. Increased ROS levels, along with decreased VEGF and AKT phosphorylation contribute to endothelial dysfunction and damaged collateral vessel formation.

#### MAPKs Signaling Pathway

MAPKs are a family of four major characterized subfamilies including ERK1/2, p38, JNK, and ERK5 (112). The MAPK signaling pathway can be activated not only by high glucose but also by glucose fluctuations. ROS and pro-inflammatory cytokines may be trigger factors. Ye et al. (113) showed that blood glucose fluctuation is involved in skin collagen metabolism by regulating the MAPK and Smad signaling pathways. P38 MAPK consists of four structurally homologous isoforms (α, β, γ, δ) and each isoform is sensitive to pharmacological inhibition differently (114). In the condition of glucose fluctuations, p38 signaling pathway activation is involved in ROS overproduction, mitochondrial dysfunction, and cell proliferation with consequent cellular damage in astroglial cells (94). Rutin, belonging to the flavonoid family, is reported to effectively reduce the vascular smooth muscle cell migration, and proliferation induced by intermittent hyperglycemia (115). Rutin can decrease ROS production, phospho-NF-κB and phosphorylation of MAPK (ERK1/2) and PI3K. Further, anti-oxidative and anti-atherosclerotic properties of rutin can be a potential treatment for diabetic patients with cardiovascular complications (115). P38 MAPK inhibitors have been used in clinical studies for cardiovascular diseases in patients with diabetes (116). However, it remains unclear that the effects of inhibiting MAPK signaling in diabetes with glucose fluctuations and further studies are needed to explore new effective treatments. NF-κB is part of ubiquitous family including several transcription factors that regulate the expression of genes in related processes such as oxidative stress, inflammation, and apoptosis (117). Zhang et al. (118) demonstrated that increased myocardial apoptosis after exposure to acute blood glucose fluctuations was associated with enhanced oxidative stress and activation of NF-κB.

### Dysregulation of microRNAs

MicroRNAs (miRNAs) are known to be short non-coding genes. Recent researches indicate that miRNAs may function as important regulators in modulation of diabetic complications. MiRNAs play significant roles in various biological processes by inducing inhibition of protein synthesis or the degradation of mRNA (119). Saito et al. (120) proposed that miRNA-200c and miRNA-141 expression levels were abundant in heart tissues of type 1 diabetic rats with glucose fluctuations, leading to an increase in ROS levels, and an aggravated ischemia/reperfusion injury state in the diabetic heart. Guo et al. (121) have found that miRNA-1273g-3p was significantly upregulated in human umbilical vein endothelial cells (fluctuations between 5 and 25 mmol/L) compared with sustained high glucose, leading to dysfunction of human umbilical vein endothelial cells. Moreover, miR-1273g-3p plays an important role in regulating cell proliferation and migration after exposure to treatment of glucose fluctuations. Further studies are expected which will explore regulation of miRNA under condition of glucose fluctuations and subsequent miRNA-based therapies may emerge as potentially important treatments for patients.

## Current Treatment Strategies Targeting Glucose Fluctuations

### Early Insulin Therapy

Several clinical studies have supported that early and appropriate insulin treatment may reduce harmful effects on β-cells induced by glucose fluctuations in both type 1 and type 2 diabetic patients (122–124). In newly diagnosed type 2 diabetes, early intensive insulin therapy has been reported to maintain extended glycaemic remission and recover β-cell function more effectively compared with oral hypoglycaemic agents (122). Moreover, Pennartz et al. (123) reported that basal treatment with long-acting insulin for patients with type 2 diabetes improved postprandial insulin secretion. A study by Bruttomesso et al. (124) in type 1 diabetes indicated that continuous subcutaneous insulin infusion reduced glucose variability and increased glycaemic control and treatment satisfaction compared with multiple daily injections with glargine. More recently, Bailey et al. (125) have found that insulin glargine 300 U/mL decreases the fluctuations in pharmacodynamic profiles and increases the uniform distribution of pharmacokinetic profiles, compared with insulin degludec 100 U/mL in a morning administration of 0.4 U/kg/day in type 1 diabetes.

### Continuous Glucose Monitoring Systems

Continuous glucose monitoring systems have been more and more increasingly used for insulin-requiring diabetic patients to monitor glucose fluctuations (126). Continuous glucose monitoring systems help diabetic patients in regulating their lifestyle and medication adjustments (127, 128). Collectively, continuous glucose monitoring seems to be an important tool with appropriate patient education to improve glycaemic stability for patients with type 1 and type 2 diabetes mellitus (129, 130).

Data from several clinical studies showed that implantable insulin pump therapy contributed to the improvement of controlling overall quality of blood glucose concentrations in diabetes (131). Moreover, administration of insulin via insulin pump together with continuous glucose monitoring improves glucose fluctuations more than conventional pump therapy in poorly controlled type 1 diabetic patients (132).

## Conclusion

In summary, glucose fluctuations appear as an important risk factor in the diabetic population. More and more clinical and experimental studies have been conducted to explore the molecular mechanisms of glucose-fluctuation-induced damaging effects. Excessive oxidative stress and inflammation which seems resultant from glucose fluctuations is involved in the development and progression of diabetic complications. Moreover, ROS can be a vital factor to triggering the activation of downstream signaling pathways, including PKC, AKT, NF-κB, and MAPK signaling pathways such to eventually cause various kinds of damaging effects in different target cells and tissues. Some miRNAs are also involved in regulation on the state of glucose fluctuations and may also have potential for use in clinical treatment. Hence, continued research that helps to further understand glucose-fluctuation-related molecular mechanisms likely will be important for developing new clinical treatments in the future.

## Author Contributions

Z-YZ and L-FM wrote the initial drafts. L-LQ, NW, M-MQ, and Y-MZ edited the manuscript. R-XW, S-PD, and YW revised the review and finalized the last version of the article. All authors checked and approved the submitted version.

### Conflict of Interest Statement

The authors declare that the research was conducted in the absence of any commercial or financial relationships that could be construed as a potential conflict of interest.

## Abbreviations

AKT: Protein kinase B
DR: Diabetic retinopathy
ERK1/2: Phosphorylation of MAPK
GA: Glycoalbumin
GPx: Glutathione peroxidase
GSK-3β: Glycogen synthase kinase-3β
HbA1c: Hemoglobin A1c
H2O2: Hydrogen peroxide
MAPK: Mitogen-activated protein kinase
MiRNAs: MicroRNAs
NADPH: Nicotinamide adenine dinucleotide phosphate
NOS: Nitric oxide synthases
•${\text{O}}_{2}^{-}$: Superoxide
•OH: Hydroxyl radical
PKC: Protein kinase C
PQS: Panax quinquefolius saponi
PTEN: Phosphatase and tensin
ROS: Reactive oxygen species
SOD: Superoxide dismutase
Trx: Thioredoxin
Txnip: Trx interacting protein
UCP2: Uncoupling protein 2
VEGF: Vascular endothelial growth factor
β-cells: Pancreatic islet Beta cells.
